# Diagnosis and Treatment of Cerebral Syphilitic Gumma: A Report of Three Cases

**DOI:** 10.3389/fnins.2018.00100

**Published:** 2018-02-27

**Authors:** Xuefei Shao, Di Qiang, Yinhua Liu, Quan Yuan, Jin Tao, Bihua Ji

**Affiliations:** ^1^Department of Neurosurgery, Yi-Ji Shan Hospital, Wannan Medical College, Wuhu, China; ^2^Department of Dermatology and STD, Yi-Ji Shan Hospital, Wannan Medical College, Wuhu, China; ^3^Department of Pathology, Yi-Ji Shan Hospital, Wannan Medical College, Wuhu, China; ^4^Department of Imaging, Yi-Ji Shan Hospital, Wannan Medical College, Wuhu, China

**Keywords:** neurosyphilis, cerebral gumma, operation, diagnosis, treatment

## Abstract

Cerebral syphilitic gumma is very rare and is often pathologically confirmed following surgery. This study reports three patients with cerebral syphilitic gumma. The first case was a 62-year-old man who was admitted to our hospital due to speech arrest for 10 hours. Head MRI showed a nodular signal shadow with a significant enhancement and a significant centerline shift. He subsequently received surgery, and cerebral syphilitic gumma was confirmed by postoperative pathology. The second patient was a 66-year-old man who was admitted to our hospital due to complaints of gradually decreasing right eye vision and headache for nearly 50 days. Enhanced MRI at admission indicated irregular clumping of high-signal mixed with low-signal foci on the frontal lobe. Subsequently, he was operatively treated and was confirmed to have cerebral syphilitic gumma by postoperative pathology. The third patient was a 37-year-old man who was admitted to our hospital due to dizziness for approximately 15 days. Head MRI indicated a slightly abnormal lamellar and longer T1, T2 signal shadow on the left side. He did not receive surgery, and his symptoms disappeared after anti-syphilitic treatment. Hence, we recommend a critical interpretation of preoperative imaging data, understanding the unique changes that arise in the brain that can be detected through imaging, and an analysis of the patient history and laboratory tests to re-evaluate the value of surgery, with the ultimate goal of performing a stabilizing treatment for cerebral syphilitic gumma.

## Introduction

Syphilis is an ancient disease that has been increasing in prevalence in recent years. According to the most recent estimation of the WHO, 17.7 million individuals worldwide aged 15–49 years had syphilis in 2012, with an estimated 5.6 million new cases every year (Newman et al., 2015). In China, an epidemiological study was performed on syphilis cases reported from 31 provinces, autonomous regions, and municipalities between 2000 and 2013. The reported syphilis incidence increased yearly from 6.43 per 100,000 person-years in 2000 to 32.86 per 100,000 person-years in 2013, with an average annual growth rate of 13.37% (Gong et al., 2014). Syphilis is a complex systemic disease caused by the bacterium *Treponema pallidum*. Gumma, also known as gummy tumor, is more common in the late stages of syphilis and is highly destructive. In the early stage, it is a deep, subcutaneous nodule that gradually grows and adheres to the skin. The central site gradually softens, ulcerates, and releases viscous, gum-like pus; hence, it is named gumma. Cerebral syphilitic gumma is rare and is often ignored during diagnosis. Because cerebral syphilitic gumma causes typical clinical symptoms and the lesions exhibit diverse characteristics on imaging, the lesions can be easily confused with brain tumors. For these reasons, syphilitic gumma often cannot be diagnosed before surgery and can only be pathologically confirmed after the operation (Ances et al., 2005; Darwish et al., 2008; Li et al., 2012; Ventura et al., 2012; Huo et al., 2013; Shi et al., 2017; Zhang et al., 2017). This study reported three cases of cerebral syphilitic gumma. All patients were treated in our hospital, two who were confirmed by postoperative pathology, and one who was cured by regular anti-syphilitic treatment. All the study participants signed a written informed consent form, and the study was approved by the ethics committee of Yi-Ji Shan Hospital of Wannan Medical College.

**Figure 1:**
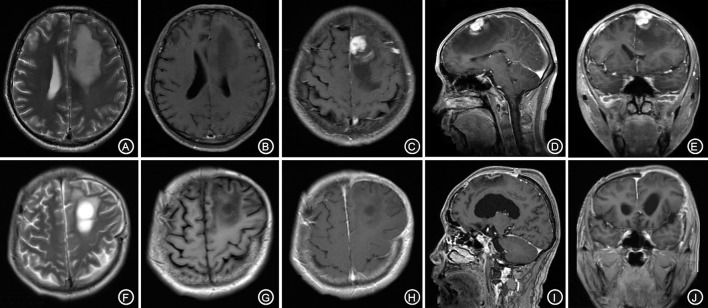
The left forehead adjacent to the cerebral falx shows a nodular equal or slightly longer T1, T2 signal **(A,B)**, surrounded by edema around the shadow. The lesion is connected to the wide base of the adjacent meninges. The adjacent cerebral convolution was an arc-shape under pressure. The foci are obviously enhanced after enhancement, and the adjacent meninges is thickened and enhanced **(C–E)**. The irregular long T1, T2 signal shadow **(F,G)** indicates a point linear enhancement shadow and no edema signal shadow **(H–J)**.

A 62-year-old male patient was admitted to our hospital due to speech arrest for 10 hours. At admission, he had clear consciousness, no nausea or vomiting, no limb convulsions, and no incontinence. Physical examination revealed no rash on the skin or mucous membranes, no tumefaction on superficial lymph nodes, bilateral isocoria with an average pupil diameter of 3.0 mm, normal light reflex, soft neck, normal cardiac rhythm, no obvious pathological murmur, and normal limb muscle strength. Head CT showed a low-density area in the left frontal lobe and ventricular compression on the same side. Head MRI showed a slightly long T1 and a long T2 nodular signal shadow adjacent to the left cerebral falx as well as a slightly high T2-FLAIR and DWI signal, with a size of approximately 1.7 × 1.8 × 1.3 cm, surrounded by edema. Additionally, the lesion was connected with the adjacent wide meningeal base, the adjacent cerebral convolution was accurate under the compression, and the adjacent lesions and meninges were thickened and enhanced after enhancement (Figures 1A–E).

**Figure 2:**
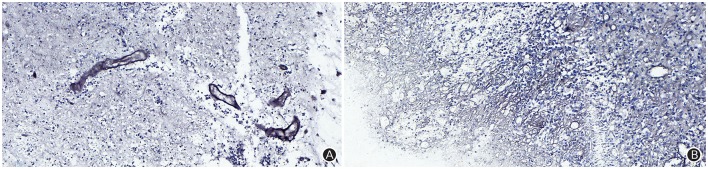
The Warthin-Starry staining (magnification: ×400) results indicated infiltration of plasma cells, lymphocytes and neutrophils, and a sleeve-like infiltration of lymphocytes around blood vessels, but did not identify *Treponema pallidum* **(A)**; The argyrophilic staining (magnification: ×400) results indicated coagulation necrosis, infiltration of lymphocytes and plasma cells, and epithelial hyperplasia in small endangium, but did not identify *Treponema pallidum* **(B)**.

The patient received symptomatic treatments to reduce the intracranial pressure. Because of the significant centerline shift and compression of the ventricle, the patient underwent resection of the occupying lesion present in the left frontal region under general anesthesia. During the operation, the arachnoid was thickened at the lesion site, with apparent nodular-like changes, had a dark red and white color, had a slightly tough texture and an ordinary blood supply, was closely connected to the sagittal sinus and had unclear boundaries with the surrounding normal brain tissues. Due to high intraoperative pressure, his bone flap was removed. Postoperative pathology showed infiltration of many plasma cells, lymphocytes, and neutrophils. The lymphocyte infiltration was sleeve-like around the blood vessels (Figure 2A). The patient underwent the reactive rapid plasma reagin circle card test and the *Treponema pallidum* particle agglutination (TPPA) test. The results were as follows: cerebral blood WBC (2 × 10^6^/L), GLU (5.28 mmol/L), Cl (119 mmol/L), negative RPR, and positive TPPA. The symptoms were relieved after anti-syphilitic treatment. At re-examination after 6 months, the patient reported that symptoms such as headache and dizziness had disappeared; however, these symptoms were not relieved after the operation (Figures 1F–J).

**Figure 3:**
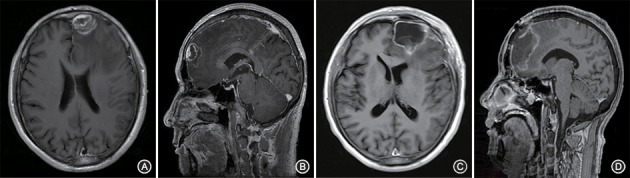
Enhanced MRI shows irregular masses lumped together mixed with high- and low-signal foci on the left frontal lobe. The border is less clear and is surrounded by a large low-signal shadow. The adjacent brain parenchyma is compressed, the lateral ventricle is compressed and deformed, and the central structure is slightly right-biased **(A,B)**. At the follow-up 10 days after surgery, the left frontal lobe still presented with an annuliform high signal, the anterior border seemed to have a high nodular signal, and the lateral ventricle was still compressed and deformed **(C,D)**.

**Figure 4:**
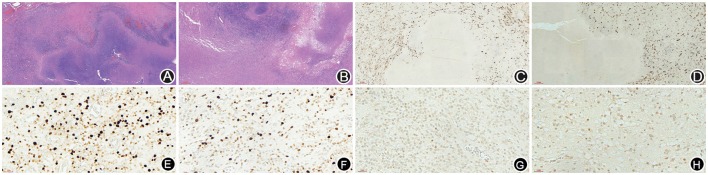
HE staining of case 1 and case 2 showed that the center of syphilis was necrotic with infiltration of a large number of inflammatory cells and glial proliferation in the periphery **(A,B)**; GFAP staining indicated a small amount of glial proliferation around necrotic foci **(C,D)**, Ki67 staining showed a higher proliferative activity around the necrotic lesions **(E,F)**, and P53 staining showed negative peripheral P53 **(G,H)**.

**Figure 5:**
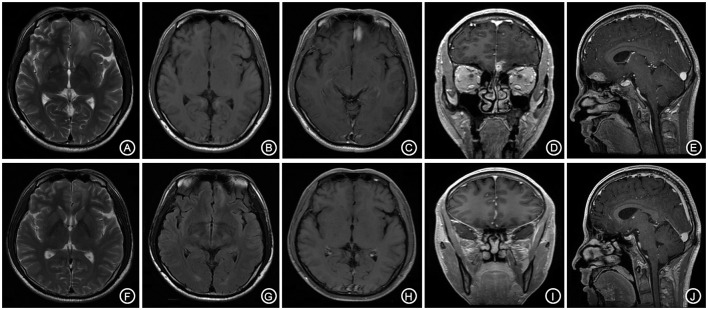
The bilateral frontal lobes showed a slightly longer and abnormal T1, T2 signal shadow with the lesion mainly on the left side **(A,B)**. The enhanced lesion is located adjacent to the left interhemispheric cistern and is patchy-shaped and obviously enhanced, with the adjacent meninges being slightly thickened and enhanced **(C–E)**. The follow-up after anti-syphilitic treatment indicated that the abnormal signal on the left frontal lobe was significantly absorbed in T1, T2 and enhanced scan **(F–J)**.

A 66-year-old male patient was admitted to our hospital with complaints of gradually decreasing right eye vision and headache for nearly 50 days. Physical examination indicated a conscious state, normal left eye vision, and no light perception in the right eye, with anisocoria and a pupillary diameter of approximately 3.0 mm. The left pupil diameter was approximately 3.5 mm, and the left eye exhibited a light-sensitive reaction. The patient had a soft neck, no resistance, and normal limb muscle strength and tension. Enhanced MRI at admission indicated irregular clumping of high-signal mixed with low-signal foci on the frontal lobe, which had an unclear border and was surrounded by a large, low signal shadow with a size of approximately 2.5 × 2.0 × 2.6 cm. The adjacent brain parenchyma and lateral ventricle were compressed and deformed, and the central structure was slightly right-biased (Figure 3). A craniotomy was performed. During the operation, the arachnoid was thickened at the lesion site, which was white and had a slightly tough texture and an ordinary blood supply. This was closely connected with the sagittal sinus and had unclear boundaries with the surrounding normal brain tissue. The rapid pathological examination indicated that these signs were inflammatory changes. Examination of the blood and cerebrospinal fluid indicated positive TPPA (+) and positive RPR (+) tests. The patient was administered with anti-syphilitic treatment. Pathological examination indicated argyrophilic staining, with no finding of *T. pallidum* (Figure 2B). The 10-day post-surgery MRI indicated an annular high signal on the left frontal lobe and a nodular high signal at the anterior border. Additionally, the adjacent brain parenchyma and lateral ventricle were compressed and deformed, with the central structure slightly right-biased (Figures 3C,D). HE staining of case1 and case 2 showed that the center of syphilis was necrotic with infiltration of a large number of inflammatory cells and glial proliferation in the periphery (Figures 4A,B); GFAP staining indicated a small amount of glial proliferation around netrotic foci (Figures 4C,D), Ki67 staining showed a higher proliferative activity around the necrotic lesions (Figures 4E,F), and P53 staining showed negative peripheral P53 (Figures 4G,H). A 37-year-old male patient was admitted to our hospital due to dizziness for approximately 15 days. At admission, he exhibited no vomiting or convulsions. Physical examination indicated normal cognitive function, normal consciousness, no yellow discoloration of the body skin or mucous membranes, no swelling of the superficial lymph nodes, bilateral isocoria with an average pupil diameter of 3.0 mm, normal light reflex, a soft neck, clear lung breath sounds, and a normal cardiac rhythm with no obvious pathological murmur. Head MRI indicated a slightly abnormal lamellar and longer T1, T2 signal shadow on the left side. After enhancement, the lesion adjacent to the left anterior interhemispheric cistern showed patchy enhancement, and the adjacent meninges were slightly thickened and enhanced (Figures 5A–E). Examination of the blood at admission showed white blood cells (14.4 × 10^9^/L), a neutrophil rate of 78%, and positive blood TP-Ab. Examination of the cerebrospinal fluid indicated protein (97.3 mg/dl), white blood cells (84 × 10^6^/L), RPR positivity, and TPPA positivity. The patient was transferred to the Department of Dermatology for anti-syphilitic treatment, which included a 14-day course of IV penicillin (2.4 million U every 4 h) followed by a 3-week course of intramuscular injections of benzathine penicillin (2.4 million U, once per week) (Workowski and Bolan, 2015). At the 4-month follow-up, normal blood indexes, normal biochemical indexes and cerebrospinal fluid indexes, negative RPR and negative TPPA were observed. The MRI re-examination showed that the abnormal signal of the left frontal lobe was significantly better than the previous images (Figures 5F–J).

## Discussion

Syphilitic gumma is a local inflammatory response of arteries or their surrounding tissues at the cerebral dura mater or cerebral pia mater caused by the invasion of *T. pallidum* into the central nervous system, which causes granulomatous changes and interstitial nerve syphilis. Neurosyphilis can be presented in a variety of forms, including acute and chronic meningitis and brain and spinal cord parenchymatous disease. The disease is often misdiagnosed in the early stage because of a lack of remarkable imaging characteristics. In 1999, Takeshima et al. reported one case of cerebral syphilitic gumma (Takeshima et al., 1999). Here, the patient was misdiagnosed with malignant lymphoma preoperatively, and only after histopathology was performed postoperatively was the patient confirmed to have cerebral syphilitic gumma. In 2013, Yoon et al. (Gürses et al., 2007) reported a resected left frontal lobe with pathologically diagnosed cerebral syphilitic gumma. The reasons for misdiagnosis of syphilis gumma are as follows: (1) syphilis gumma is rarely observed in the clinic, and medical staff lack knowledge about the disease; (2) the information contained in the patient history is not detailed, and patients and their families tend to hide the patient history; and (3) the imaging characteristics of neuronal syphilitic gumma are not reliable. The first two reasons are subjective factors, and the correct analysis of patient imaging data may prevent unnecessary surgery.

Although patient imaging cannot be used to confirm cerebral syphilitic gumma, it can be used to further correlate the characteristics of cerebral syphilitic gumma. MRI examination of cerebral syphilitic gumma also has the following manifestations. Contrast-enhanced MR imaging shows a rounded lesion located in the cerebral cortex and subcortex; caseous necrosis of the lesion center has low signal foci or mixed normal and low signal foci. This is surrounded by the low signal area caused by a large area of edema and has an occupying effect; Gd-DTPA enhanced scans reveal that the lesions have an irregular annular enhancement, with the edge being intersected with the surrounding meninges at an obtuse angle; and finally, the meninges and cranial nerves around the lesions are thickened and enhanced. Cerebral syphilitic gumma has a similar imaging appearance to other diseases including inflammatory granuloma, brain metastases, and malignant meningioma, which have significantly enhanced nodules with necrosis at the center of the lesions and are surrounded by large areas of edema. The most characteristic distinguishing feature is that cerebral syphilitic gumma encroaches on and is closely related to the meninges; however, the edge of the lesion often intersects with the surrounding meninges at an obtuse angle. The extracranial lesions are primarily located outside the cerebral parenchyma. The MRI of the above three cases showed that the simple nodules at the bottom of the anterior cranial fossa and on the brain surface adjacent to the cerebral falx were significantly enhanced and had a slightly longer T1, T2 signal. These findings were similar to those for meningioma; however, after careful analysis, necrotic lesions were found at the nodule center of the syphiloma, which was surrounded by granulation tissue, lymphocytes, epithelial cells, and multinucleated giant cells. The outermost layer tends to present with vasculitis due to glial cell proliferation and angiogenic infiltration of mononuclear cells. Large areas of edema usually surround the lesions and have high signal foci at T2WI or mixed signal foci with a normal, high and low signal. The low signal is due to the cheese-like granuloma produced by the macrophages and is surrounded by extensive edema. The nodule is obviously enhanced, which differs from the common meningioma (Gürses et al., 2007). Lin et al. (2009) used PET/CT to measure the presence of fluoride deoxyglucose (F18-2-fl u-2-deoxy-D-glucose, FDG) metabolism in the brain to diagnose cerebral syphilitic gumma, which is discriminated through the dispersion of tension (Soares-Fernandes et al., 2007).

Although cerebral syphilitic gumma is not confirmed by pathology, it can be diagnosed by clinical and laboratory examinations. Syphilitic gumma also shows a unique meningeal tail sign of meningiomas, indicating that cerebral syphilitic gumma is reversible. The first patient in this study required removal of the bone flap due to obvious intraoperative brain swelling. The patient had repeated headaches and dizziness caused by obvious postoperative brain edema. Upon examination of the cerebrospinal fluid and after the diagnosis was confirmed by pathology, the conditions of the patient improved after conventional anti-syphilis treatment. There is no doubt regarding performing surgery on acute intracranial hypertension patients. For patients without malignant intracranial hypertension who are diagnosed with neurosyphilis by combined analysis of their history and related laboratory test, it is recommended to administer a non-surgical treatment such as anti-syphilis treatment, which may avoid unnecessary surgeries. Fargen et al. (2009) analyzed 156 cases of syphilitic gumma, which were mostly located in the convex surface of the brain (66%), were sensitive to antibiotics, and apparently disappeared or were completely absorbed after treatment. Therefore, cerebral syphilitic gumma patients can apply this diagnostic treatment to avoid unnecessary craniotomy. The third patient in this study was found to have basic nodules that disappeared by 6 months after anti-syphilitic treatment, which also demonstrates this viewpoint.

The diagnosis of neurosyphilis requires a consideration of the patient history, clinical manifestations, syphilis serology, cerebrospinal fluid examination, imaging, and other physiological changes. The neurosyphilis diagnosis is mainly based on clinical manifestations of patients and laboratory tests of serum and cerebrospinal fluid. Patients who are suspected to have neurosyphilis should undergo syphilis serological tests, such as TPPA and RPR, biochemical tests, and TPPA and RPR detection of the cerebrospinal fluid. Neurosyphilis is usually a complication of late syphilis, which generally occurs 3–20 years after a syphilis infection but may also occur as early as 2 years after the infection.

The first goal in treating neurosyphilis is to clear the *T. pallidum* invading the central nervous system. For some patients who have had irreversible damage to the nervous system, the progression of the disease must be stabilized to promote the greatest potential of functional recovery. Second, the serum should be normalized, and a non-*T. pallidum* antigen test should be performed.

## Author contributions

XS and DQ: write the article; YL and QY: collect the materials; JT and BJ: view the whole paper.

### Conflict of interest statement

The authors declare that the research was conducted in the absence of any commercial or financial relationships that could be construed as a potential conflict of interest.
